# Challenges in personalised management of chronic diseases—heart failure as prominent example to advance the care process

**DOI:** 10.1186/s13167-016-0051-9

**Published:** 2016-01-30

**Authors:** Hans-Peter Brunner-La Rocca, Lutz Fleischhacker, Olga Golubnitschaja, Frank Heemskerk, Thomas Helms, Thom Hoedemakers, Sandra Huygen Allianses, Tiny Jaarsma, Judita Kinkorova, Jan Ramaekers, Peter Ruff, Ivana Schnur, Emilio Vanoli, Jose Verdu, Bettina Zippel-Schultz

**Affiliations:** 1 Heart Failure Clinic, Department of Cardiology, Maastricht University Medical Center, PO Box 5800, 6202AZ Maastricht, The Netherlands; 2 Fleischhacker GmbH, Schwerte, Germany; 3 EPMA, Brussels, Belgium; 4 RIMS bvba, Overijse, Belgium; 5 German Foundation for the Chronically Ill, Fürth, Germany; 6 Sananet Care BV, Sittard, Netherlands; 7 Linköping University, Linköping, Sweden; 8 Medical Faculty Pilsen, Pilsen, Czech Republic; 9 Exploris AG, Zürich, Switzerland; 10 sense.ly, San Francisco, USA; 11 Mulimedica SPA, Milano, Italy; 12 Medtronic Iberica SA, Madrid, Spain

**Keywords:** Predictive preventive personalised medicine, Cardiovascular disease, Heart failure, Chronic diseases, Care processes, Health economics, Future care, Communication and interaction

## Abstract

Chronic diseases are the leading causes of morbidity and mortality in Europe, accounting for more than 2/3 of all death causes and 75 % of the healthcare costs. Heart failure is one of the most prominent, prevalent and complex chronic conditions and is accompanied with multiple other chronic diseases. The current approach to care has important shortcomings with respect to diagnosis, treatment and care processes. A critical aspect of this situation is that interaction between stakeholders is limited and chronic diseases are usually addressed in isolation.

Health care in Western countries requires an innovative approach to address chronic diseases to provide sustainability of care and to limit the excessive costs that may threaten the current systems. The increasing prevalence of chronic diseases combined with their enormous economic impact and the increasing shortage of healthcare providers are among the most critical threats. Attempts to solve these problems have failed, and future limitations in financial resources will result in much lower quality of care. Thus, changing the approach to care for chronic diseases is of utmost social importance.

## Background

Chronic diseases are by far the leading causes of mortality and morbidity in Europe [1]. Together, heart disease, stroke, cancer, chronic respiratory diseases, diabetes mellitus and other chronic diseases represent 2/3 of all death causes. The prevalence of chronic diseases is rising due to changes in lifestyle, increasing healthcare standards and therefore lifespan and is most relevant to the ageing population. Besides adverse effects on quality of life, chronic diseases pose a serious economic burden as 75 % of all healthcare costs are comprised of chronic diseases [2].

A significant contribution to the burden is the co-concurrence of multiple chronic diseases (co-morbidities), which are increasingly frequent [3] and are present in 1/3 of the adult population. Chronic diseases should not be treated separately [4], but caregivers often are not capable to consider the interaction of multiple factors relevant for individual care plans. Also, clinical evidence merely focuses on single diseases and fails to provide evidence on how to manage multiple diseases as patients with co-morbidities are usually excluded from large clinical trials. Multiple diseases often require involving several caregivers. However, hospital care, ambulatory specialist care and primary care are subdivided into numerous entities, based mainly on medical specialty [5]. The existing gaps in coordination and communication between the caregivers hamper the care process [6] and are associated with medical errors [7]. Hence, providing such patients with optimal and integrated care is a major challenge.

### Heart failure—a prominent example of chronic diseases

Heart failure is a chronic, debilitating disease *and* represents a frequent co-morbidity in patients with other primary diseases. Heart failure care is often further complicated by general frailty and a need for highly organised and integrated care. Considering its complexity, heart failure care involving multiple specialists is an ideal, representative model to change the strategy of care for multiple chronic diseases.

The incidence (390 per 100,000 person years [8]) of heart failure is alarmingly high, and its prevalence is steadily rising. Approximately 2 % of the population in Western societies suffers from heart failure, and this figure will rise to 3 % by 2025 (i.e. >20 million people in Europe) [9]. The percentage rises sharply with age to approximately 10 % of the population aged 75 years or older or even almost 20 % in those aged 85 years or more [1]. This high overall prevalence is partly caused by the increase in unhealthy lifestyle, such as poor diet and lack of physical activity of the general population. Paradoxically, a further increase of heart failure prevalence is unavoidable, not only due to the ageing of the population but also due to decreased mortality by better treatment of underlying diseases such as myocardial infarction. Treatment reduces acute mortality, but leaves patients with damaged hearts resulting in heart failure [10], specifically if the unhealthy lifestyle is not corrected. In addition, associated diseases, such as hypertension and atrial fibrillation, are expected to increase in the future [11]. Finally, treatment of heart failure improves lifespan, but lacks to effectively cure it, further contributing to the increase in prevalence, which may represent a rising economic burden.

Despite improved treatment, heart failure is still associated with debilitating symptoms, high hospitalisation rates and poor prognosis [12]. This compares unfavourably with other chronic diseases with an average 5-year survival rate after first heart failure admission of only 35–50 % [13, 14]. In-hospital care is frequent, often lengthy and costly (61 % of costs consist of in-hospital care [15]), accounting for approximately 2 % of total expenditure on health care in Western countries [9]. Within only 3 months after discharge from hospital, 24 % of patients are readmitted [10], highlighting the challenges in heart failure care. Reducing hospitalisation is highly warranted to reduce costs particularly in patients with multiple co-morbidities as (re-)hospitalisation is often caused by other morbidities. Research on healthcare processes in heart failure in three countries (the Netherlands, Belgium, Germany) shows that care is not optimally organised and presents significant overlap, insufficient communication and poorly defined pathways/strategies in care [16]. Thus, there is a significant room for streamlining care to further reduce costs.

### Heart failure and co-morbidities: complicate diagnosis, treatments and follow-up

Risk factors for heart failure are overlapping with other chronic diseases, and therefore, patients with heart failure frequently have many co-morbidities. More than 40 % of heart failure patients have five or more co-morbidities (e.g. atrial fibrillation, hypertension, diabetes, COPD, renal failure, rheumatic disorders, stroke, depression, cancers), while almost none is free of any co-morbid condition [17, 18]. The frequent presence of co-morbidities complicates diagnosis, treatment and follow-up and is an important reason for inadequately organised care. The cumulative number of drugs for these patients increases the risk of interactions and adverse effects. Co-morbidities may interfere with treatment effects of heart failure medication [19], and most of them are associated with worse prognosis [20]. Hence, co-morbidities significantly complicate care and patients with multiple diseases should be treated holistically and in a personalised way, but *in reality*, this is not the case. Current practice hardly addresses an integrated management of co-morbidities. Interaction and communication between patients, relatives and caregivers as well as alignment between caregivers in different settings are often poor, which may be one of the most important reasons for insufficient care [16].

### Care process to be redesigned

Aspects such as diagnosis, treatment, follow-up and organisation all play important roles in the care process to optimise outcome with similar difficulties observed across different countries in Europe.

#### Diagnosis

Inaccurate or late diagnosis of heart failure, underlying reason(s) and complications as well as co-morbidities result in poor quality of life and outcome due to poor prediction of personalised needs, resulting in inadequate treatment and lack of preventing progression. Hence, there is a need for guidance in determining which diagnostic tests should be performed and in adequately interpreting results.

Symptoms of heart failure, like dyspnoea, are often non-specific and do not discriminate heart failure from other diseases. Especially in primary care where patients present at early stages and symptoms are mild, heart failure is difficult to diagnose [21]. Approximately 50 % of heart failure cases are diagnosed by the general practitioners (GPs) [22]. Most other patients are diagnosed at the time of the first hospitalisation. As diagnosis is difficult, part of the patients are wrongly or not diagnosed. Evaluation of the diagnostic process shows that in both primary and secondary care, basic investigations mentioned in the guidelines are often not performed [10, 23]. All these shortcomings can lead to hospitalisation or even death [10]. Importantly, a substantial number of these hospitalisations as well as progression of disease(s) could be prevented by early and accurate identification and/or patient monitoring.

#### Treatment

Treatment plans for heart failure patients are not adequately personalised and do not consider co-morbidities. This may cause under- or overtreatment, resulting in side effects and poor outcome. There is an urgent need for personalised treatment plans taking all patient’s characteristics into account.

Effective therapies for heart failure are often not utilised in a safe, timely, equitable, patient-centred and efficient manner [24]. This is partly caused by insufficient awareness of relevant research evidence due to the rapidly changing therapeutic field. Significant variation is observed in prescribing heart failure medication in different care settings. Although guidelines recommend treatment plans for specific conditions, they lack to give advice on comprehensive treatment plans for multiple chronic diseases. Still, considering the increasing number of medications used, those personalised plans are crucial as overall, more than half of the patients use four or more different medications [25]. Besides the lack of personalised treatment planning, patient adherence and persistence of prescribed treatment is poor. Patients are confused by the high number of medications and the transition of care setting (in-hospital medication is administered while at home the patient is responsible).

The increasing complexity, assisted by developments in the information and communication technology (ICT) sector, resulted in decision-support systems for heart failure care. The main goal of such systems is to improve the clinical decision making process by providing the necessary knowledge to procure patients with optimal care. Algorithms generate recommendations to support healthcare professionals. Still, their usage is hampered by different challenges for successful implementation. (1) Integration into clinical setting is difficult since care pathways and “ownership” are hardly defined; (2) comprehensibility in terms of ease of use and language is often not given; (3) systems are mostly used as add-ons to existing care instead of substitution of care; (4) semantic and technical interoperability of existing systems as well as with electronic patient records is a major challenge that is not yet resolved; (5) involvement of patients (or their family) regarding self-management is basically absent. Importantly, these systems are usually limited to one specific disease, failing to address co-morbidities and personalised needs of patients. Not surprisingly, there is no evidence yet that such systems would improve outcome and there is currently no implementation of decision support for personalised treatment. Capabilities and effectiveness of various remote monitoring systems have been reviewed recently, showing these limitations [26].

#### Chronic management

Lack of follow-up strategies and patient involvement results in poor outcomes despite increasing costs. Hence, there is a need to develop and implement personalised patient support strategies.

Once a patient is diagnosed and treatment started, follow-up strategies involving patients should be developed. Patients have important responsibilities including adherence to treatment, making significant lifestyle changes, monitoring themselves and reacting in case of problems. The importance of this is often recognized, but seldom implemented [27], and interventions are often not integrated as a whole, but focussed on separate parts of treatment. Self-management is an active cognitive process undertaken by patients to manage their own chronic diseases [28]. It encompasses the adoption of numerous practices, follow-up with caregivers and emotional management [24]. However, adoption of each of these individual care components may be difficult for patients as they may be hard to understand, seen as inacceptable intrusion in daily life and may interfere with recommendations given related to co-morbidities.

Different programmes have been set up to support patients such as structured telephone support, self-management support programmes and tele-monitoring, but implementation is limited [29]. These programmes usually are not sufficiently personalised, particularly not addressing specific co-morbidities. There is evidence of efficacy of self-management programmes [30], but evidence is of mixed quality and does not adequately take account programme complexity and heterogeneity [31]. Moreover, results in clinical practice may be substantially different as found in Canada [32]. Possibly, disease managed programmes or approaches may work in some groups of patients, but there may be also a large group of patients that is not responding optimally or not at all. Tele-monitoring and structured telephone support of patients with heart failure may reduce hospitalisation and mortality rates [33]. However, two recent large randomised controlled trials failed to improve outcome [34, 35]. Invasive monitoring may be more efficient, but again focussed on heart failure only and due to the invasive nature applicable to a limited part of patients only [36]. In clinical practice, programmes failed to show consistent improvements and it is unclear which parts are clinically important [37]. Usually, recorded parameters are limited and give little insights in the actual condition of individual patients [26]. Thus, there are limitations regarding implemented strategies to support patients during their long-term follow-up, particularly regarding self-management. Moreover, decision support still is in its infancies. In part, this may be related to the fact that short-term outcome measure and surrogate parameters are not sufficiently solid to support personalised decision making.

#### Interactions

Lacking interactions between stakeholders leads to poor comprehensibility of the care process. There is a need to facilitate communication including patients to personalise management.

**Fig. 1:**
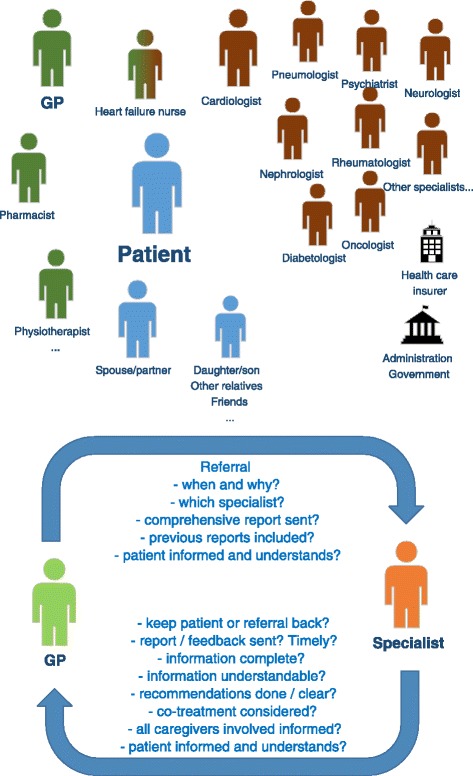
Most important stakeholders in the care of a HF patient. Involvement of specialists other than cardiologist as required (not a complete list). *Blue* patient and family, *green* primary care, *brown* secondary/tertiary care

All stakeholders of an individual patient should collaborate and interact appropriately. In the care for patients with chronic diseases, many stakeholders are involved (Fig. 1). However, communication and interaction between patients, their relatives and caregivers as well as among caregivers have received little attention so far, are poorly defined and are largely insufficient. We found that this is one of the most important reasons why care of chronic diseases is not optimal, both with respect to efficiency and costs [16].

Communication between patients and caregivers is often poor and not oriented at the individual patient [38]. Patients see up to 15–23 caregivers within both primary and secondary care annually [39]. Patients are poorly informed and do not understand their condition. Some may not even know that they have been diagnosed with heart failure. They often are not aware of the roles and responsibilities of the different caregivers. The lack of communication between patients and their caregivers results not only in lower quality of care but also quality of life, associated with poor adherence to treatment and required changes in lifestyle. Obviously, this forms a major hurdle towards personalised management of patients with chronic diseases.

**Fig. 2:**
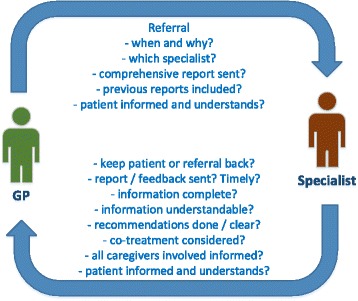
Required information exchange between two caregivers

Unfortunately, interaction between different caregivers involved in the care for different co-morbidities is often absent. Sometimes specialists are unaware of the presence of co-morbidities and the co-medication a patient is taking [6]. Moreover, information provided by specialists to GPs is often late and poorly understood by the GP. A multidisciplinary approach and a central coordinator are often lacking. In clinical reality, even the simple communication loop between two caregivers (e.g. GP and cardiologist, Fig. 2) is usually incomplete, which becomes more evident the more caregivers are involved.

To improve these shortcomings, there has been a shift in heart failure management towards a multidisciplinary approach in recent years. This shift has led to several randomised controlled trials of multidisciplinary, organised and/or managed care [40]. Most of these approaches applied to specialist personnel mainly within multidisciplinary teams. In some of these programmes, formal links were established between healthcare professionals including not only cardiology care, which resulted in fewer hospital readmissions than routine care patients in some studies [39, 41]. However, in a real-world setting, multidisciplinary care in Canada was found to slightly reduce mortality, but at the cost of even a significant increase in heart failure-related admissions [32].

Attempts to implement multidisciplinary heart failure care have only been partly successful in a few European countries. An important reason for the poor implementation might be that most programmes are stand-alone, focusing on limited aspects of heart failure care. Lack of standardisation, limited evidence of cost-effectiveness as well as lack of flexibility and acceptance by healthcare providers are additional reasons for poor implementation.

#### Evidence-based care

Clinical evidence retains patients with co-morbidities from optimal care. There is a need for evidence-based personalised care to address co-morbidities of individual patients.

To facilitate care for heart failure patients, the European Society for Cardiology (ESC) and other authorities developed evidence-based guidelines [12]. These guidelines are complex and adherence to them is regrettably poor [42]. They cover recommendations for heart failure, but they are still very limited with respect to care for co-morbidities.

Multiple treatment trials have been performed resulting in evidence-based clinical guidelines. These trials applied precisely defined inclusion and exclusion criteria usually not including patients with co-morbidities. Thus, the majority of patients are not properly represented in the large treatment trials [43]. Hence, evidence is limited for patients seen in daily practice, i.e. with multiple chronic diseases. Various systematic reviews of guidelines concluded that evidence in older patients with co-morbidities is poor [43–45] and that adherence to current guidelines in that specific subset of patients may even have undesirable effects [46]. Various case-control studies found an increased risk of severe adverse drug reactions in patients at older age and particularly with multiple co-morbidities [47–49], but there is a lack of clinical recommendations integrating different guidelines to support personalised care for patients with multiple chronic diseases.

Table 1 shows three potential and realistic patient scenarios. Scenario 1 is fully covered by the guidelines, but represents only approximately 10 % of the heart failure population. Scenario 2 gives an example of a patient with one important co-morbidity, which is largely covered by the guidelines, but leaves patients with limited treatment options for the co-morbidity. Again, this is not yet the typical heart failure patient. Scenario 3 is more frequently occurring regarding complexity of multiple chronic diseases. This scenario is not covered by guidelines. There are many patients with even more co-morbidities and more drugs. Management of such patients is challenging and not only requires good communication between caregivers, patients and their relatives but also requires prospective treatment trials addressing this important issue.

**Table 1:** Examples of different patient scenarios with similar underlying cardiac disease, depicting how the presence of co-morbidities significantly impacts treatment considerations and where guidelines fail to give guidance

Heart failure patient scenario	Presence of co-morbidities	Treatment considerations
Stage C heart failure [12] (male, 74 y, LVEF 25 %) due to coronary artery disease (CAD) with dyspnoea during exercise only, clinically stable	Scenario 1- No major co-morbidities (mild COPD, diabetes on diet only)	• Guidelines recommended medical heart failure therapy: ACE inhibitor, β-blocker, eplerenone, ev. loop diuretic; ICD placement • Guidelines recommended CAD therapy: statin, low-dose aspirin • Diabetes diet • No fluid and salt excess • Physical exercise
	Scenario 2- Arthritis as single important co-morbidity	Additional treatments required: • Anti-inflammatory drug (NSAID) might interact with ACE-inhibitor resulting in acute renal failure • Chronic NSAID use might disturb fluid balance and worsen heart failure • Patient might avoid exercise due to pain. He may benefit not only from involvement of physiotherapy but also from clear communication between cardiologist and rheumatologist to discuss individually tailored therapy and exercise plan
	Scenario 3 - Paroxysmal symptomatic atrial fibrillation - More severe diabetes - Reduced renal function (creatinine 220 μmol/l) - Iron deficiency anaemia	Additional treatments required: • Higher dose of loop diuretic, but may further worsen renal function • Amiodarone for rhythm control (might cause pulmonary fibrosis and hyper- or hypothyroidism) • Oral anticoagulation; may cause interaction with other medication! • 1 or 2 antidiabetic drugs; may cause interaction with other medication! • Iron deficiency requires search for bleeding (often gastrointestinal) and ev H_2_-blocker; may cause interaction with other medication! • Iron infusion and possibly erythropoietin due to renal failure
		Additional important aspects (incomplete list): • Patient was told by GP to drink sufficiently to improve renal function; might result in worsening heart failure. Proper instruction of patient and collaboration between GP, nephrologist, cardiologist and patient needed! • Renal failure: ACE-inhibitors and eplerenone might induce hyperkalaemia • Prescribed diet: low potassium and phosphorus on top of other diet; this complex diet might need the involvement of a dietician. • ACE-inhibitor and possibly eplerenone dosage should be adjusted and renal function closely monitored (by whom?) • Heart failure might result in worsening kidney function and vice versa, requiring a close collaboration between caregivers • Various potential interactions between drugs (this patient requires approximately 12 different drugs!) require specific attention • Adherence of the patient might be poor given the number of medication

*y* years, *LVEF* left ventricular ejection fraction, *CAD* coronary artery disease, *COPD* chronic obstructive pulmonary disease, *ICD* internal cardioverter defibrillator, *NSAID* non-steroidal anti-inflammatory drug, *GP* general practitioner

#### In summary, current care for patients with heart failure and related co-morbidities is far from being optimal, which leaves a large margin for improvement

**Fig. 3:**
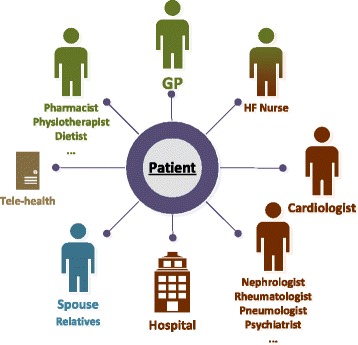
Current one-directional care in chronic diseases (*green* refers to primary care, *brown* to specialist care)

From all these issues in diagnosis, treatment, chronic care, interactions between stakeholders and available evidence for heart failure patients with co-morbidities, major underlying issues can be pointed out: firstly, there is lack of interaction, sufficient communication and knowledge exchange between patients and their caregivers as well as among caregivers with different expertise. Secondly, care is largely unidirectional from individual caregivers to patients, with virtually absent personal responsibility by the patients. Thirdly, the lack of clinical evidence for patients with multiple chronic diseases heavily hampers the care of these patients. Fourthly, ICT/tele-medicine plays so far an isolated, limited role and is used in addition to other care instead of as substitution of parts of care, and data exchange is not integrated in care (Fig. 3). Unfortunately, there is currently no framework available addressing these issues.

### Innovative PPPM approach in management of chronic diseases

Based on the considerations regarding current gaps in personalised treatment of patients with heart failure and multiple co-morbidities, several aspects of health care should be differently addressed in the future [50]. This may result not only in better treatment of individual patients with chronic diseases resulting in better outcome and quality of life, but also in better prediction of such needs and in the prevention of both progression of disease(s) and development of additional chronic disorders.

- Collaboration, interaction and communication between all persons involved in the care of chronic diseases needs to be substantially improved. The patient must be central in his/her management system, as person-centred care in heart failure seems to be promising [51]. All involved caregivers must have access to the information about the patient. Therefore, central ICT and easy data exchange are important. The patient (and his/her representative if required) provides rights to access the information.
- Patient involvement for the development of new ICT platforms is crucial to define the requirements in view of the fact that the typical patient with chronic diseases is elderly with difficulties to understand and use standard ICT equipment. In order to support acceptance and later adoption of the new system, the innovation process should include not only the perspective of the patient, but also the needs of the other stakeholders in the care of chronic diseases. Constant feedback during the development process is important.
- Analysis and integration of multiple guidelines are crucial. Therefore, multidisciplinary committees should be formed to address this issue. Moreover, multidisciplinary prospective cohorts with (long-term) follow-up are needed.
- Prediction of outcome should be improved by addressing multiple chronic diseases and focusing on short-term changes that may be able to predict long-term outcome and respond to personalised treatment. This may allow steering the individualised care process more effectively and efficaciously.
- Prospective treatment trials addressing multiple chronic diseases are required.
- Progress requires not only novel and innovative technology but also an innovative and novel vision of care and health.

Obviously, this list cannot be exhaustive in covering all future needs. Also, various initiatives and projects are required to address all these issues as it is impossible to address them all together. Moreover, it may take years until significant progress is being made. However, very little attempts are being made so far in this regard and a paradigm shift is required in health care. Only then, the imminent problems in health care can be tackled to provide good quality care at affordable costs for all.

## Conclusions

Health care in Western countries requires a new innovative approach to address chronic diseases such as heart failure to provide sustainability of care and to limit the excessive costs that may threaten the current system. The increasing prevalence of chronic diseases together with their enormous economic impact and the increasing shortage of health care providers are amongst the most critical threats. Attempts to solve these problems have failed so far. Future limitations in financial resources will result in a significant reduction in the quality of care. Thus, changing the approach to care of chronic diseases is of utmost social importance. This needs not only adoption and smarter use of modern technology, but also new a vision on both care and health.

## Abbreviations

GP: general practitioner
ICT: information and communication technology
